# “Breakthrough” osmosis and unusually high power densities in Pressure-Retarded Osmosis in non-ideally semi-permeable supported membranes

**DOI:** 10.1038/srep45168

**Published:** 2017-03-23

**Authors:** Andriy Yaroshchuk

**Affiliations:** 1 ICREA, pg.L.Companys 23, 08010 Barcelona, Spain; 2 Department of Chemical Engineering, Polytechnic University of Catalonia, av. Diagonal 647, 08028 Barcelona, Spain

## Abstract

Osmosis is the movement of solvent across a membrane induced by a solute-concentration gradient. It is very important for cell biology. Recently, it has started finding technological applications in the emerging processes of Forward Osmosis and Pressure-Retarded Osmosis. They use ultrathin and dense membranes supported mechanically by much thicker porous layers. Until now, these processes have been modelled by assuming the membrane to be ideally-semipermeable. We show theoretically that allowing for even minor deviations from ideal semipermeability to solvent can give rise to a previously overlooked mode of “breakthrough” osmosis. Here the rate of osmosis is very large (compared to the conventional mode) and practically unaffected by the so-called Internal Concentration Polarization. In Pressure-Retarded Osmosis, the power densities can easily exceed the conventional mode by one order of magnitude. Much more robust support layers can be used, which is an important technical advantage (reduced membrane damage) in Pressure-Retarded Osmosis.

Osmosis was the first membrane phenomenon discovered experimentally back in the 18^th^ century^1^. It is of paramount importance for the cell biology. Recently, the process has started finding technological applications. Forward Osmosis and Pressure-Retarded Osmosis increasingly draw attention as potential technologies of choice for the pre-treatment (pre-dilution) in seawater desalination^2^, desalination of produced water and fracturing flow-back^3^, dissolution of fertilizers by using impaired water sources^4^, and energy harvesting from salinity gradients^5^,^6^ to mention just a few examples. In these processes, a highly concentrated (draw) solution is used to extract solvent (typically water) from a solution of lower osmolality through a semi-permeable membrane. The purpose is either to obtain water (like in Forward Osmosis) or to use the osmotic volume flow for energy harvesting from salinity gradients (like in Pressure-Retarded Osmosis). In the latter process, hydraulic counter-pressure is applied across the membrane, which makes possible obtaining mechanical work.

In the case of monolayer semi-permeable membranes the physical picture of osmosis is relatively simple: the osmotic flow is just proportional to the difference of osmotic pressures between the solutions separated by the membrane. However, stand-alone monolayer semipermeable membranes usually exhibit quite low osmotic flows (due to relatively large thickness) and are not suitable for technological applications. Therefore, composite and/or asymmetric membranes are used instead where an ultra-thin (∼100 *nm*) semi-permeable layer is supported by a much thicker (∼100 *μm*) porous layer. In such arrangements the solute concentration at the barrier-layer/support interface can be strongly affected by the osmotic flow itself, which is referred to as Internal Concentration Polarization. This is illustrated by Fig. 1. The Internal Concentration Polarization is a non-linear phenomenon so it can dramatically reduce the concentration difference across the barrier (active) layer, which is the driving force of the process. Depending on the trans-membrane volume flow, the Internal Concentration Polarization reduces the osmotic-pressure difference across the osmotically-active barrier layer to a larger or smaller extent so instead of being a linear function of osmotic-pressure difference between the solutions, the flow often shows a slower, logarithmic dependence on it. The Internal Concentration Polarization is essentially controlled by the diffusion permeability of support layer. Therefore, considerable effort has been devoted to the preparation of Forward Osmosis/Pressure-Retarded Osmosis membranes with as thin and loose support layers as possible^7^,^8^,^9^,^10^,^11^. This, however, can compromise the membrane mechanical stability. This is especially important in the Pressure-Retarded Osmosis where considerable (potentially, millions of Pa) hydrostatic counter-pressures are applied across the membrane. Accordingly, in the flat-sheet configuration the membranes must be supported mechanically, which is usually achieved via placing them on top of 3D polymer spacers. The thinner and looser the membrane the more probable is a membrane “collapse” into the spacer meshes probably compromising the membrane integrity^8^,^12^. On the other hand, using excessively dense spacers can considerably increase the energy cost of cross-flow pumping of dilute solution stream. The mechanical properties are also very important for hollow-fiber Pressure-Retarded Osmosis membranes^7^,^9^. Thus, research in this field has often been about a compromise between an acceptable level of Internal Concentration Polarization and the membrane mechanical properties.

This study demonstrates that for a certain kind of barrier-layer materials and operational conditions the Internal Concentration Polarization can be made negligible (irrespective of support diffusion permeability) so much thicker and more robust support layers can be used without compromising the membrane performance. This behavior is shown to occur for the so-called “leaky” (non-ideally semi-permeable) barrier layers where the solute reflection coefficient somewhat deviates from unity.

Until very recently, the Forward Osmosis/Pressure-Retarded Osmosis processes have been modelled exclusively by assuming the membrane barrier layers to be ideally semi-permeable^13^,^14^. Only in one recent study^15^ the so-called Spiegler-Kedem model^16^ was used for the simulation of performance of Pressure-Retarded Osmosis modules with “leaky” membranes. However, in this study the model was utilized only for fitting experimental data. General features of the system behavior were not explored.

In this work, we will use the standard Spiegler-Kedem model to describe the solute and solvent transfer through the barrier layer and the standard convection-diffusion equation in the support layer. In spite of simple and well-known modelling tools, our analysis reveals the existence of previously overlooked mode of osmosis in composite/asymmetric membranes we will refer to as “breakthrough” osmosis. The availability of analytical solutions will enable us to formulate simple criteria for the occurrence of this mode. We will also demonstrate that in Pressure-Retarded Osmosis the “breakthrough” mode can give rise to unprecedented power densities. Moreover, they are practically unaffected by the Internal Concentration Polarization, which represents a considerable technological advantage. Again, the analytical solutions will afford simple criteria for the maximum power density and threshold hydrostatic counter-pressure that makes the “breakthrough” mode disappear abruptly. Finally, we will demonstrate that the favorable combination of membrane transport properties is predicted by the well-known model of steric hindrance for solutes that closely match the membrane pores in size.

Below, we will concentrate on the so-called Pressure-Retarded Osmosis membrane orientation (active layer facing the more concentrated (draw) solution). The opposite (Forward Osmosis) orientation can be analyzed in a similar way but is not considered because it does not exhibit the “breakthrough” mode.

## Results

### Criteria of “breakthrough” mode

Figure 2 shows dimensionless volume flux (Péclet number in the support layer defined by equation (14) divided by the parameter *ρ* defined by equation (21)) and the solute concentration at the barrier-layer/support interface scaled on the draw-solution concentration (*c*_*i*_/*c*_*d*_) as functions of parameter *F* (defined by equation (19)). According to this definition this parameter is proportional to the osmotic pressure in the draw solution. For good Forward Osmosis membranes *F* can be around 500 at draw-solution concentration of 1 M NaCl^10^ so the range shown in Fig. 2 is realistic. For comparison, the figure also shows the case of ideally-semipermeable barrier layer.

The assumed reflection coefficient (*σ* = 0.99) is quite close to *σ* = 1 (ideally semipermeable barrier layer). At not too large *F*, those two cases practically coincide. However, at some critical value of parameter *F* (see below), they depart dramatically. In the “ideal” case (*σ* = 1), the interface concentration keeps increasing, which makes the concentration difference across the barrier layer ever smaller so the osmotic flow increases in a slow logarithmic way. In the slightly “leaky” case (*σ* = 0.99), the interface concentration has a maximum. To the right from it, this concentration starts to decrease (and the concentration difference across the barrier layer to increase), which makes the osmotic flow grow dramatically. Therefore, we refer to this behavior as the “breakthrough” mode. Below, we will see that this dramatic change in the behavior is ultimately caused by the change in the direction of solute flow through the membrane and a positive feedback.

Expectably, the most unusual behavior occurs with very thick supports because in the conventional mode the osmotic flow is practically zero in this limiting case due to a very strong Internal Concentration Polarization. On the contrary, in the “breakthrough” mode it is non-zero (and large). Remarkably, Fig. 2 shows that in this mode the osmotic flow becomes insensitive to the properties of membrane support (the curves calculated for different support properties converge). Therefore, the limiting case of infinitely thick supports considered below is applicable also for finite (and even rather small) values of parameter *ρ* once the breakthrough mode is well established.

At *ρ* → ∞ (but *σ* ≠ 1), equation (13) (see Methods) can be transformed to


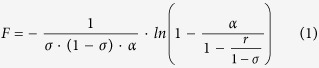


where parameter *α* ≡ 1 − *c*_*i*_/*c*_*d*_ is the dimensionless concentration difference across the barrier layer. Figure 2 shows that for slightly “over-critical” values of *F* at not too small *ρ*, the interface concentration is close to the draw concentration so this parameter remains small. Therefore, one can expand the right-hand side of equation (1) in *α* to obtain.


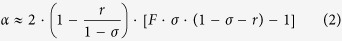


For *α* in equation (2) to be positive (as it should because the interface concentration is always lower than the draw concentration), parameter *F* has to be larger than.


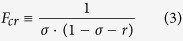


For *F*_*cr*_ to be finite and positive the dimensionless feed concentration must be smaller than 1 − *σ*. Therefore, with slightly “leaky” barrier layers (reflection coefficient very close to one) the “breakthrough” mode occurs only with very dilute feed solutions. Thus for instance, if *σ* = 0.99 the feed solution must be, at least, more than 100 times more dilute than the draw solution for the “breakthrough” mode to occur.

From equation (1) one can see that


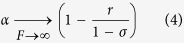


The corresponding limiting dimensionless volume flux is 
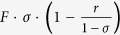
, that is a linear function of draw osmotic pressure. This is dramatically different from the logarithmic increase occurring for ideally-semipermeable membranes.

### Solute flux

A fundamental difference between the ideally semi-permeable and “leaky” membranes is that in the latter some convective solute transfer is possible. This increases in a roughly linear way with the transmembrane volume flow. Besides, in osmotic processes, the direction of convective solute flow is opposite to the diffusion. Due to this, in “leaky” membranes the total solute flux (being the sum of diffusion and convection components) can change sign. This can be seen from equation (13). Initially, the interface concentration increases super-linearly with the draw concentration. At the same time, the exponential factor by it becomes ever smaller. Calculations show that sooner or later this leads to change of sign of the numerator, which becomes negative and the solute flow turns positive (directed in the same way as the volume flow). For the change of sign to occur the solute transport in the barrier layer has to be dominated by the (partially-coupled) convection. Due to the complete convective coupling in the support the solute transport there is dominated by the convection, all the more so. This is illustrated by Fig. 3.

In the mode of sufficiently strong convection, the material balance of solute at the interface is controlled by the balance between the solute brought to the interface convectively through the support and the solute withdrawn from this interface due to the convective flow of solute through the barrier layer. At dominant convection, the flux of solute toward the interface can be approximated by *c*_*f*_ · *J*_*v*_. The flux of solute away from it is roughly equal to *c*_*i*_ · (1 − *σ*) · *J*_*v*_. Thus, if the feed concentration is lower than the product of interface concentration and one minus reflection coefficient the solute withdrawal can be stronger than the delivery. (Note that just on the threshold to the “breakthrough” mode, the interface concentration can reach values that are quite close to the draw concentration.) Accordingly, the interface concentration tends to decrease. This makes somewhat weaker the convective solute withdrawal through the barrier layer but simultaneously causes an increase in the concentration difference across the barrier layer and in the osmotic flow. Quantitative analysis shows that overall this makes the convective solute withdrawal ever stronger and the decrease in the interface concentration ever more pronounced with increasing draw concentration. Thus, there is a positive feedback that explains the avalanche (or breakthrough) nature of this mode. This feedback disappears only when the interface concentration approaches the feed concentration so the volume flux becomes a linear function of parameter *F* (see Fig. 2). As shown above, this mode can occur as long as *r* < 1 − *σ*. Notably, the solute flux changes direction even when *r* > 1 − *σ*. However, in this case, the convective solute delivery through the support to the interface is stronger than the convective solute withdrawal from the interface across the barrier layer. The positive feedback does not occur, the interface concentration keeps increasing with the trans-membrane volume flow and the “breakthrough” mode does not develop.

Despite the positive solute flux in the “breakthrough” mode a perpetuum mobile is not possible in this system (as well as elsewhere). Due to the co-directional solvent transfer (simultaneous dilution) the solute concentration in the draw solution tends to decrease rather than increase with time. In other words, the so-called chemical solute flux into the draw solution defined as


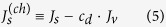


remains negative even though the solute flux is positive.

### Pressure-Retarded Osmosis with supported “leaky”· membranes

In Pressure-Retarded Osmosis, a positive hydrostatic-pressure difference is applied between the draw and feed solutions. This partially retards osmotic flow and ultimately makes possible energy harvesting. The “breakthrough” mode features unprecedentedly high trans-membrane volume flows. Therefore one can also expect unusually high power densities in Pressure-Retarded Osmosis. However as shown above, this mode occurs only at sufficiently large trans-membrane volume flows that cause the reversal in the direction of solute flow in the case of “leaky” membranes. The counter-pressure decreases the volume flow so the “breakthrough” mode is more probable to occur at lower counter-pressures. Moreover, at a certain counter-pressure one can expect this mode to disappear. This is illustrated by Fig. 4 showing volume flux (scaled on its maximum value occurring at no counter-pressure) vs. counter-pressure.

Remarkably, at larger relative diffusion resistances of support layer, there is a range of counter-pressures where the trans-membrane volume flow becomes a multivalued function of counter-pressure. There are three branches. The upper one corresponds to the “breakthrough” mode, the lower one corresponds to the conventional mode, and the intermediate branch is probably unstable. We speculate that within this range of counter-pressures the system behavior exhibits hysteresis. When the counter-pressure is increased from zero, the volume flow changes along the upper branch until the point of infinite negative derivative is reached. At larger counter-pressures, there is only one branch, namely, the conventional (lower) one. Therefore, the volume flux has to “jump” down to this branch. When the counter-pressure is decreased from close to the draw osmotic pressure, initially, the volume flow changes along the lower branch just because no other branch is available. We hypothesize that until the lower-pressure inflection point is reached, the system remains on the lower branch and “jumps” up to the upper branch at this point. Further analysis is needed to explore the stability of these solutions. Nonetheless, even without such analysis one can see that for not too thick support layers, the lower-pressure inflection point is located at not too low counter-pressures so there is a considerable counter-pressure range where the “breakthrough” mode is the only solution.

Figure 5 shows the corresponding dimensionless power densities defined by equations (34) and (35), which are proportional to the product of trans-membrane volume flow and hydrostatic counter-pressure. Since the power density is proportional to the volume flow it also can be a multivalued function of counter-pressure. The power density is unusually large only at the upper branch. It is possible that with counter-pressures between the lower-pressure and higher-pressure inflection points (see Fig. 5) the upper branch is unstable. However, as discussed above, at not too large values of parameter *ρ* (dimensionless *S*-parameter) there is a sufficiently large range of counter-pressures (between zero and the lower-pressure inflection point) where the upper high-power-density branch is the only solution.

At lower counter-pressures, the system behaves as if there were practically no Internal Concentration Polarization (compare with the case of *ρ* = 0). Accordingly, the power densities are essentially larger than in the conventional mode. The difference is especially pronounced for larger values of *ρ* (thicker and/or denser supports). Thus for instance, for 1 M NaCl draw solution and by using the hydraulic permeability of the hollow-fiber PRO membrane reported in ref. 10 (hollow fiber B, *A* = 9.2 · 10^−12^ *m*/(*s* · *Pa*)) in the “breakthrough” mode we obtain the power density of about 50 *W/m*^2^ at the point of maximum whereas in the conventional mode (at *ρ* = 0.1) we get only about 7 *W/m*^2^ (note that for the membrane studied in ref. 10 the value of this parameter was different). Besides, in the conventional mode the maximum is located at an approximately 50% higher counter-pressure, which is known to be a technical challenge because of possible membrane damage at higher counter-pressures (see above). In the “breakthrough” mode the power density is proportional to the square of draw concentration (see equation (33) below) whereas in the conventional mode the increase is essentially slower due to the stronger Internal Concentration Polarization at higher draw concentrations. For example with 2 M NaCl, the maximum power density can be estimated at above 200 *W/m*^2^ in the “breakthrough” mode whereas it is only 15 *W/m*^2^ in the conventional mode.

Figure 5 shows that up to the higher-pressure inflection point the behavior of power density is very well captured by the limiting case of ρ ≫ 1. (Strictly speaking, this is true only at sufficiently large *F* and not too small *ρ* but just this combination of parameters is of principal interest in practical terms.) In this limiting case, one can easily obtain simple analytical criteria for the optimal counter-pressure (corresponding to the maximum power density) and the critical counter-pressure (corresponding to the higher-pressure inflection point) (see Methods). Figure 6 shows those two characteristic counter-pressures as functions of parameter *F*. The optimal counter-pressure remains below 50% of draw osmotic pressure, which is beneficial in terms of mechanical stress the membrane is exposed to.

### Steric-Hindrance Model for Closely-Matching Spheres in Cylindrical Pores

For the “breakthrough” mode to occur the membrane should combine some deviations from ideal semi-permeability with quite low solute diffusion permeability. Interestingly, such a behavior is predicted by the well-known model of steric hindrance for closely-matching spheres in narrow pores. Figure 7 shows the ratio of convection and diffusion hindrance factors calculated for a spherical solute in a cylindrical pore by using the best available equations provided in ref. 17.

For closely-matching solutes (*λ* ≤ 1), this ratio increases dramatically, which indicates that one can have some deviations from the ideal semi-permeability (due to a not too strong convective hindrance) and a strongly reduced solute permeability (due to the much stronger diffusion hindrance) at the same time. Of course, this is just the limiting case where the applicability of this macroscopic model of steric hindrance to solutes of molecular dimensions is far from evident. Nonetheless, this model may still reflect some features of reality.

## Conclusions

In this study, we used standard Spiegler-Kedem model to investigate theoretically Forward Osmosis and Pressure-Retarded Osmosis in bilayer (asymmetric/composite) membranes with non-ideally semi-permeable active layers (“leaky” membranes). Our analysis has revealed that:

- “Leaky” asymmetric/composite membranes can exhibit previously overlooked mode of “breakthrough” osmosis where the negative impact of Internal Concentration Polarization is strongly reduced.
- This mode occurs only in the Pressure-Retarded Osmosis configuration (barrier layer facing more concentrated draw solution), at sufficiently large draw concentrations, sufficiently low solute permeability of barrier layer and for quite dilute feed solutions.
- In Pressure-Retarded Osmosis, this mode can give rise to unprecedentedly high power densities. These are possible for membranes with quite large *S*-parameters; this can be a considerable technological advantage in terms of membrane robustness and lifetime.
- In the “breakthrough” mode of Pressure-Retarded Osmosis, dependences of trans-membrane flow on counter-pressure can become multi-valued within certain counter-pressure ranges; this can give rise to hysteresis on the flow-pressure characteristics.
- The combination of membrane properties required for the breakthrough mode to occur can be predicted by the classical model of steric hindrance for solutes closely matching membrane pores in size.

## Methods

**Figure 1:**
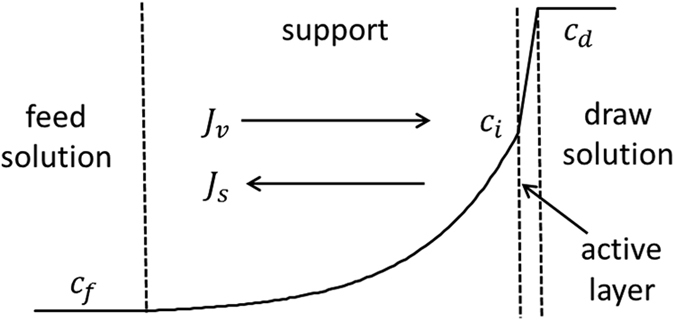
Schematics of Forward Osmosis in supported membranes; *J*_*v*_ is the volume flow, *J*_*s*_ is the solute flow; *c*_*f*_, *c*_*i*_, *c*_*d*_ are the solute concentrations in the feed solution, at the barrier-layer/support interface and in the draw solution, respectively.

**Figure 2:**
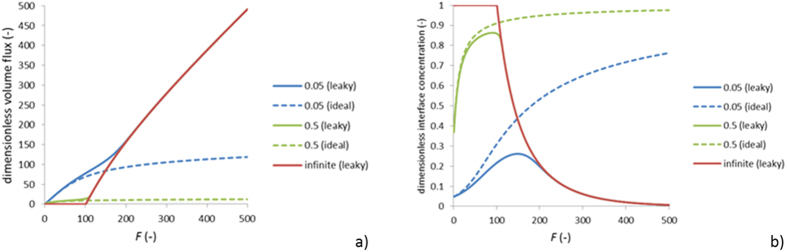
Dimensionless osmotic flow across (**a**) and dimensionless interface concentration in (**b**) supported “leaky” (*σ* = 0.99) and ideally semipermeable (*σ* = 1) membrane; zero concentration in the feed solution; the legends indicate the values of parameter *ρ* (dimensionless *S* parameter defined by equation (21)); parameter *F* is proportional to the draw osmotic pressure and defined by equation (19).

The bi-layer system as well as a typical concentration profile (in the conventional mode) is shown in Fig. 1.

The solute and solvent transport equations in the barrier layer read this way


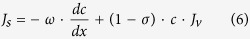



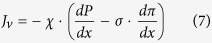


where *c* is the reference (virtual) solute concentration (Reference (virtual) solution is defined as such that could be in thermodynamic equilibrium with a given point inside the membrane; see ref. 18 for more detail), * ω* is the solute permeability, *σ* is the solute reflection coefficient, *J*_*s*_ is the solute flux, *J*_*v*_ is the volume flux, 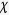 is the mechanical permeability, *P* is hydrostatic pressure, *π* is osmotic pressure (both in the reference solution). In the limiting case of *σ* = 1, equations (6 and 7) reduce to the solution-diffusion model^19^.

If the material constants are independent of either coordinate or concentration (Spiegler-Kedem model^16^), equation (7) can be integrated over the barrier-layer thickness


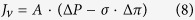


where


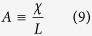


is the hydraulic permeance of the barrier layer (as customary, we neglect the hydraulic resistance of the porous support).

At constant material constants, equation (6) can be integrated, too


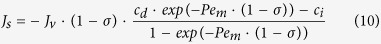


where


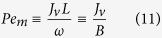


*L* is the barrier-layer thickness, *c*_*d*_ is the solute concentration in the draw solution, *c*_*i*_ is the solute concentration at the barrier-layer/support interface. In the support


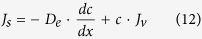


where *D*_*e*_ is the effective diffusion coefficient of the solute (accounting for the finite porosity and pore tortuosity). Equation (12) can also be easily integrated.


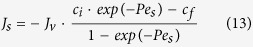


where


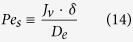


*δ* is the support thickness, *c*_*f*_ is the solute concentration in the more dilute (feed) solution.

Due to the one-dimensionality of the fluxes, both solute flux, *J*_*s*_, and volume flux, *J*_*v*_, are the same in the barrier layer and the support. Therefore, from equation (10) and equation (13), we obtain


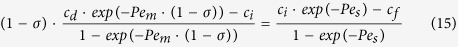


For simplicity, we neglect the external concentration polarization (existence of unstirred solution layers close to the membrane surfaces). In this case, the draw- and feed-side concentrations at the membrane surfaces, *c*_*d*_ and *c*_*f*_, are known. The Péclet numbers *Pe*_*m*_ and *Pe*_*s*_ are directly proportional to the transmembrane volume flow, which is given by equation (8).

In the case of non-retarded osmosis (Δ*P* = 0), by additionally assuming the solution to be ideal, we can relate the trans-membrane volume flow to the concentration difference across the barrier layer.


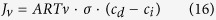


where *v* is the salt stoichiometric coefficient. By using the definitions of Péclet numbers of equation (11) and equation (14) after some identical transformations we obtain this transcendental equation for the dimensionless concentration difference across the barrier layer,


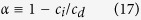



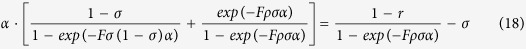


where


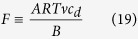



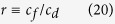



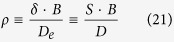


and the so-called *S*-parameter is the thickness of solution layer having the same diffusion permeance as the support layer (*D* is the solute diffusion coefficient in the bulk solution).

At *σ* = 1, equation (18) reduces to this


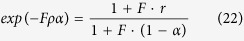


which is the well-known result obtained for ideally-semi-permeable barrier layers^20^. From equation (15) we can obtain this inverse relationship between the Péclet number in the support (or any other parameter directly proportional to the trans-membrane volume flow) and the driving force of the process (parameter *F*).


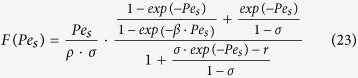


where we denoted


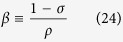


### Concentration profiles

**Figure 3:**
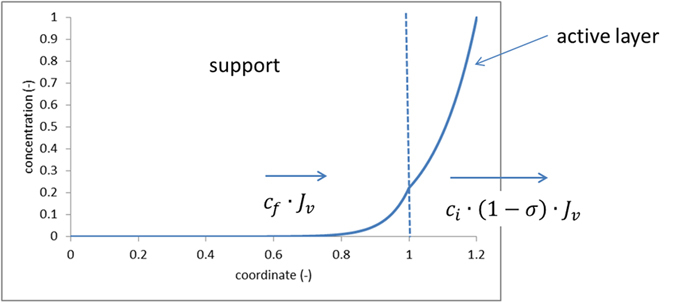
Schematics of solute-concentration profile in the “breakthrough” mode (not to scale); *c*_*f*_ · *J*_*v*_ is the convective solute flux in the support, *c*_*i*_·(1 − *σ*) · *J*_*v*_ is the convective solute flux in the barrier layer (see text for more detail).

**Figure 4:**
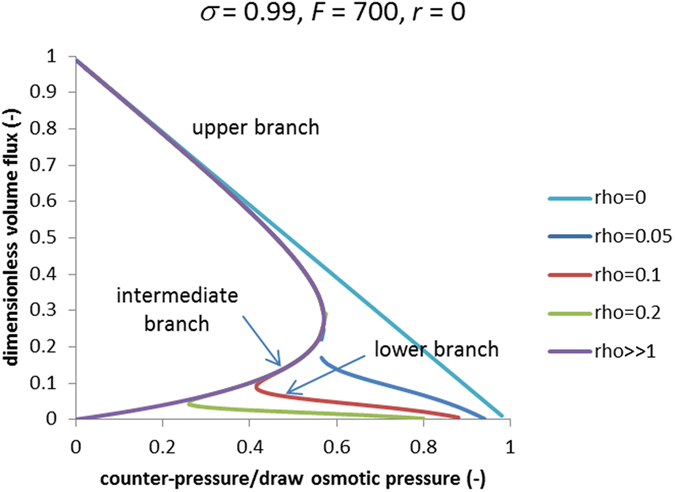
Dimensionless trans-membrane volume flux vs. dimensionless hydrostatic counter-pressure in PRO.

The concentration profile shown in Fig. 3 was calculated by using these equations.

In the barrier layer


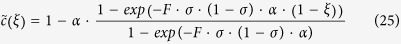


In the support:


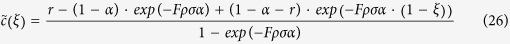


where the dimensionless virtual concentration, 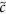, is scaled on the draw concentration, *c*_*d*_, *ξ* is the dimensionless coordinate scaled on the thickness of the corresponding layer.

### Pressure-Retarded Osmosis with supported “leaky” membranes

Being formulated in terms of trans-membrane volume flow, equation (15) is applicable to the Pressure-Retarded Osmosis case, too. One just has to modify the expression for the volume flow, which in this case reads


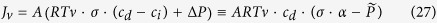


where


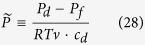


is the dimensionless hydrostatic pressure difference between the draw and feed solutions scaled on the osmotic pressure in the draw solution. In deriving equation (28) we assumed (as customary) that the hydraulic resistance of support layer is negligible compared to the barrier layer.

By using equation (27), we can define the Péclet numbers featuring in equation (15).


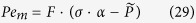



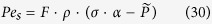


These can be substituted to equation (15) to obtain


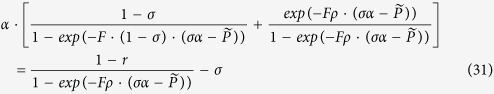


For a given dimensionless hydrostatic-pressure difference, this is a transcendental equation in *α* (dimensionless concentration difference across the barrier layer). In the limiting case of ideally-semipermeable membrane (*σ* = 1) equation (31) reduces to


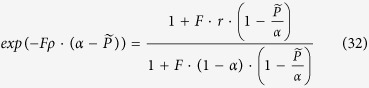


At 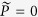 (unimpeded osmosis) equation (31) reduces to equation (18).

The power density is the product of volume flow and hydrostatic counter-pressure.


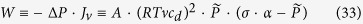



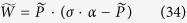


where we have defined the dimensionless power density as


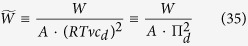


 Π_*d*_ is the osmotic pressure in the draw solution and *α* should be found from equation (31).

**Figure 5:**
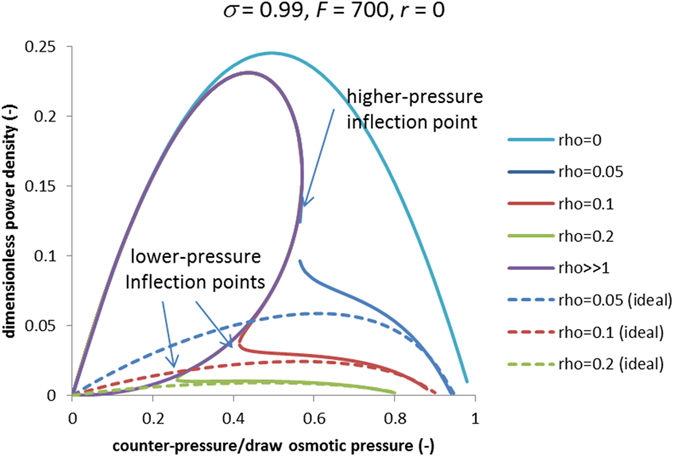
Dimensionless power density in Pressure-Retarded Osmosis vs. dimensionless hydrostatic counter-pressure; solid lines show the “leaky” case, dashed lines correspond to the “ideal” case; for *ρ* = 0 (no support layer) the “leaky” and “ideal” cases practically coincide (only the “leaky” case is shown).

**Figure 6:**
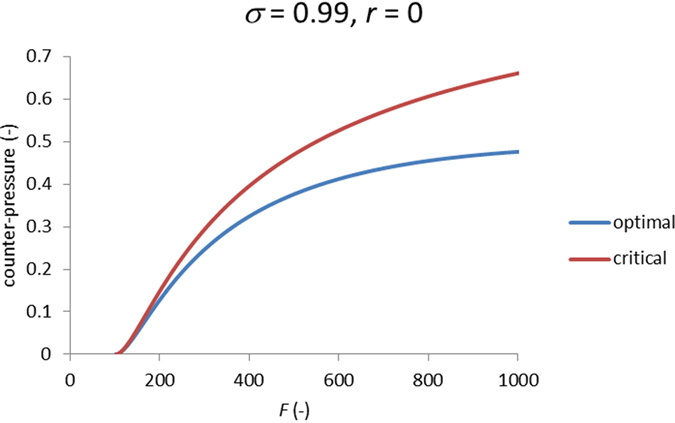
Dimensionless (scaled on draw osmotic pressure) optimal and critical counter-pressures vs. *F* in the limiting case of *ρ* ≫ 1.

**Figure 7:**
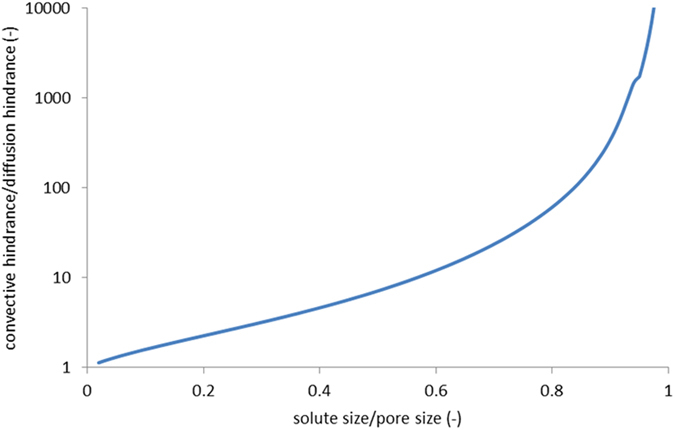
Ratio of convection and diffusion hindrance factors for the model of hydrodynamic hindrance of movement of a hard sphere in a cylindrical pore.

Above we have seen (see Fig. 5) that around the maximum power density the “breakthrough”-mode branch is very well captured in the limiting case of thick supports (*ρ* → ∞). In this limiting case equation (31) reduces to


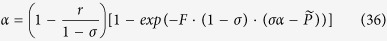


This can be explicitly resolved in the dimensionless counter-pressure to obtain this parametric relationship between the dimensionless power density and the dimensionless counter-pressure.


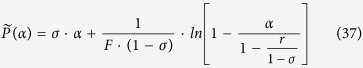



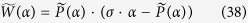


With regard to the range of variation of parameter *α*, in the “breakthrough” mode the solute-concentration difference across the barrier layer increases with the trans-membrane volume flow. A counter-pressure reduces this flow so the largest value of parameter *α* (dimensionless concentration difference across the barrier layer) occurs when 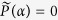. Therefore, for the maximum value of parameter *α* from equation (37), we obtain


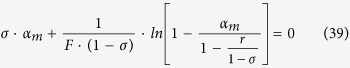


When the counter-pressure increases, at a certain point the trans-membrane volume flow becomes too small to support the breakthrough mode. At *ρ* → ∞, in the conventional mode, *α* = 0 due to the infinitely strong Internal Concentration Polarization. Therefore, the lower limit of variation of *α* is zero.

### Maximum power density and higher-pressure inflection point

Figure 5 shows that the zone around the maximum power density is very well captured in the limiting case of *ρ* ≫ 1. Moreover, even the location of higher-pressure inflection point is well captured, too. By using the parametric relationships of equations (37) and (38), it is easy to obtain equations for their locations. The criterion for the maximum power density is 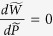, which gives rise to this transcendental equation for the value of parameter *α* corresponding to the maximum power density.


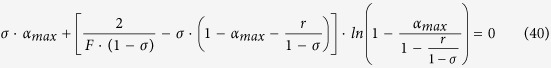


The maximum power density occurs at the optimal counter-pressure 
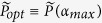
 and is equal to *W(α*_*max*_).

The criterion of the higher-pressure inflection point is 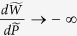. The corresponding counter-pressure can be shown to be


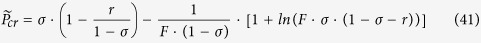


In the limiting case of *ρ* ≫ 1, the lower-pressure inflection point is located at zero counter-pressure. Therefore, the analysis of its location requires considering finite values of *ρ* and the use of full equation (31).

## Additional Information

**How to cite this article**: Yaroshchuk, A. “Breakthrough” osmosis and unusually high power densities in Pressure-Retarded Osmosis in non-ideally semi-permeable supported membranes. *Sci. Rep.* **7**, 45168; doi: 10.1038/srep45168 (2017).

**Publisher's note:** Springer Nature remains neutral with regard to jurisdictional claims in published maps and institutional affiliations.

## References

[b1] Nollet J. A. Leçons de physique expérimentale. (Hippolyte-Louis Guerin & Louis-Francios Delatour, 1748).

[b2] Altaee A., Mabrouk A. & Bourouni K. A novel Forward osmosis membrane pretreatment of seawater for thermal desalination processes. Desalination 326, 19–29 (2013).

[b3] Coday B. D. & Cath T. Y. Forward Osmosis: Novel Desalination of Produced Water and Fracturing Flowback. J. Am. Water Works Assoc. 106, E55–E66 (2014).

[b4] Phuntsho S. . Fertiliser drawn forward osmosis desalination: the concept, performance and limitations for fertigation. Rev. Environ. Sci. Bio/Technology 11, 147–168 (2011).

[b5] Kim Y. C. & Elimelech M. Potential of osmotic power generation by pressure retarded osmosis using seawater as feed solution: Analysis and experiments. J. Memb. Sci. 429, 330–337 (2013).

[b6] Helfer F., Lemckert C. & Anissimov Y. G. Osmotic power with Pressure Retarded Osmosis: Theory, performance and trends – A review. J. Memb. Sci. 453, 337–358 (2014).

[b7] Chen Y., Setiawan L., Chou S., Hu X. & Wang R. Identification of safe and stable operation conditions for pressure retarded osmosis with high performance hollow fiber membrane. J. Memb. Sci. 503, 90–100 (2016).

[b8] She Q. . Fabrication and characterization of fabric-reinforced pressure retarded osmosis membranes for osmotic power harvesting. J. Memb. Sci. 504, 75–88 (2016).

[b9] Chou S., Wang R. & Fane A. G. Robust and High performance hollow fiber membranes for energy harvesting from salinity gradients by pressure retarded osmosis. J. Memb. Sci. 448, 44–54 (2013).

[b10] Zhang S. & Chung T. S. Minimizing the instant and accumulative effects of salt permeability to sustain ultrahigh osmotic power density. Environ. Sci. Technol. 47, 10085–10092 (2013). 23941367 10.1021/es402690v

[b11] Yip N. Y., Tiraferri A., Phillip W. A., Schiffman J. D. & Elimelech M. High Performance Thin-Film Composite Forward Osmosis Membrane. Environ. Sci. Technol. 44, 3812–3818 (2010). 20408540 10.1021/es1002555

[b12] Hickenbottom K. L., Vanneste J., Elimelech M. & Cath T. Y. Assessing the current state of commercially available membranes and spacers for energy production with pressure retarded osmosis. Desalination 389, 108–118 (2016).

[b13] Mccutcheon J. R. & Elimelech M. Modeling Water Flux in Forward Osmosis: Implications for Improved Membrane Design. AIChE J. 53, 1736–1744 (2007).

[b14] Cath T. Y. . Standard Methodology for Evaluating Membrane Performance in Osmotically Driven Membrane Processes. Desalination 312, 31–38 (2013).

[b15] Attarde D., Jain M. & Gupta S. K. Modeling of a forward osmosis and a pressure-retarded osmosis spiral wound module using the Spiegler-Kedem model and experimental validation. Sep. Purif. Technol. 164, 182–197 (2016).

[b16] Spiegler K. S. & Kedem O. Thermodynamics of hyperfiltration (reverse osmosis): criteria for efficient membranes. Desalination 1, 311–326 (1966).

[b17] Dechadilok P. & Deen W. M. Hindrance Factors for Diffusion and Convection in Pores. Ind. Eng. Chem. Res. 45, 6953–6959 (2006).

[b18] Yaroshchuk A. E. Osmosis and reverse osmosis in fine-porous charged diaphragms and membranes. Adv. Colloid Interface Sci. 60, 1–93 (1995).

[b19] Wijmans J. G. & Baker R. W. The solution-diffusion model: a review. J. Memb. Sci. 107, 1–21 (1995).

[b20] Yaroshchuk A. Influence of osmosis on the diffusion from concentrated solutions through composite/asymmetric membranes: Theoretical analysis. J. Memb. Sci. 355, 98–103 (2010).

